# Predicting spatial familiarity by exploiting head and eye movements during pedestrian navigation in the real world

**DOI:** 10.1038/s41598-025-92274-4

**Published:** 2025-03-07

**Authors:** Markus Kattenbeck, Ioannis Giannopoulos, Negar Alinaghi, Antonia Golab, Daniel R. Montello

**Affiliations:** 1 Research Unit Geoinformation, TU Wien, 1040 Vienna, Austria; 2 Energy Economics Group, TU Wien, 1040 Vienna, Austria; 3 Department of Geography, UC Santa Barbara, Santa Barbara, CA 93117 USA

**Keywords:** Human behaviour, Computer science

## Abstract

Spatial familiarity has seen a long history of interest in wayfinding research. To date, however, no studies have been done which systematically assess the behavioral correlates of spatial familiarity, including eye and body movements. In this study, we take a step towards filling this gap by reporting on the results of an in-situ, within-subject study with $N=52$ pedestrian wayfinders that combines eye-tracking and body movement sensors. In our study, participants were required to walk both a familiar route and an unfamiliar route by following auditory, landmark-based route instructions. We monitored participants’ behavior using a mobile eye tracker, a high-precision Global Navigation Satellite System receiver, and a high-precision, head-mounted Inertial Measurement Unit. We conducted machine learning experiments using Gradient-Boosted Trees to perform binary classification, testing out different feature sets, i.e., gaze only, Inertial Measurement Unit data only, and a combination of the two, to classify a person as *familiar* or *unfamiliar* with a particular route. We achieve the highest accuracy of $89.9\%$ using exclusively Inertial Measurement Unit data, exceeding gaze alone at $67.6\%$, and gaze and Inertial Measurement Unit data together at $85.9\%$. For the highest accuracy achieved, yaw and acceleration values are most important. This finding indicates that head movements (“looking around to orient oneself”) are a particularly valuable indicator to distinguish familiar and unfamiliar environments for pedestrian wayfinders.

## Introduction

Understanding the needs of users during route following is key to developing effective personalized wayfinding assistance systems (see, e.g., Giannopoulos^1^ for a discussion of the importance of these systems). User needs depend greatly on the state of their spatial cognition during route following, while they engage in wayfinding through the environment. Any such system needs, therefore, to be able to infer the user’s current state of spatial cognition in-situ. A prime example of such an important state of spatial cognition, known to considerably impact wayfinding behavior, is spatial familiarity (see, e.g., Alinaghi et al.^2^, Muffato et al.^3^, or Ralph et al.^4^). Ideally, a personalized wayfinding assistance system should be able to accurately derive the degree of familiarity its user has in-situ, in an unobtrusive manner. As familiarity is known to have a considerable impact on wayfinding behavior (see e.g. Kim et al.^5^ or Guo et al.^6^), assessing this state of spatial cognition is one promising pathway to achieve personalized wayfinding assistance. In order to facilitate this, however, determining a person’s level of familiarity should be done based on sensor data in order to be both, unobtrusive and acknowledging the change of familiarity over time. Here, we report on a study which takes a first step towards this goal. We collected data during an in-situ wayfinding task carried out by pedestrians ($N=52$) by monitoring their location, head movements, and gaze behavior while they walked two routes, one in a familiar area and another in an unfamiliar area. Online data collection with the same group of participants had initially determined routes that were either familiar or unfamiliar for each person. Following an exploratory approach, we then use this behavioral gaze/head/body movement data to conduct a number of machine-learning experiments to understand which features stemming from which sensory data allow us to most clearly distinguish between a person traveling through familiar versus unfamiliar areas. We show that Inertial Measurement Unit (IMU) data provide a promising means to make this distinction, and head movements are particularly suitable for this purpose.

## Related work

Given our focus here, we review two strands of related work. We start by briefly reviewing the conception of environmental familiarity and its assessment; we show that despite some ambiguity in the literature and lack of clarity in theorizing about it, the concept of environmental familiarity has often been applied to various research questions concerning pedestrian wayfinding in outdoor environments. We then review work on exploiting gaze behavior and IMUs for research on spatial cognition by means of Machine Learning (ML). Gaze recording has been applied to a variety of research questions for some time, and the use of IMUs in wayfinding research has recently seen increasing interest, too.

### Conceptions of familiarity

Reviewing work on familiarity in spatial learning in 1982, Acredolo^7^ pointed out that scholars either study the consequences of familiarity (e.g., the increase in knowledge of landmark locations) or aim to understand which variables contribute to the development of a sense of familiarity. Her review provided evidence for the multi-faceted nature of familiarity, a nature that has been reinforced multiple times. For example, Gale et al.^8^ identified four dimensions of familiarity in two human-subject studies using a set of well-known places: knowing where a place is located, recognizing a place based on its visual appearance, recognizing the name of a place, and the interaction frequency by visiting a place or passing-by. Aggregating their data across all places, Gale and colleagues report that the dimensions are considerably correlated yet still distinct. This finding does not hold equally well if looking at the data per place, in which case correlations between interaction frequency and any of the remaining three dimensions drop^8^. Gale and colleagues take this as evidence that the understanding of the term *spatial familiarity* varies across individuals. Kitchin^9^ identified familiarity as one of nine variables which impact the development of a cognitive map. He proposed four different measures of familiarity (recall, visual recognition, locational accuracy, and amount of experience) and stressed the concept’s spatial and aspatial components (i.e., a landmark’s location vs. its appearance). Of these, “experience” has been used frequently in the literature as a proxy for, or measure of, familiarity. For example, Piccardi et al.^10^ used the duration of residence in a particular town as a measure of familiarity when they developed a scale for assessing spatial cognitive style and familiarity. Other authors have used experience, in particular, as a measure for route familiarity, often neglecting the fact that the frequency with which a route is taken versus being given the opportunity to learn a route as part of a study are two different ways to conceptualize (and measure) route familiarity^11^. It also ignores the fact that two people with the same amount of experience at a place may still know the place to different degrees (see e.g. Montello^12^).

### Studying familiarity and its impact

Research on environmental familiarity has focused on a variety of problems, ranging from evacuation (see e.g. Song et al.^13^) to pointing accuracy (see e.g. Lehnung et al.^14^) and landmark selection (see e.g. Li et al.^15^). Despite an increased interest in indoor environments recently^5,16,17^, most work has considered outdoor environments, and our own focus here is on outdoor spaces, specifically on pedestrians following routes in outdoor spaces (whether virtual or real).

Researchers in various disciplines, including psychology (cognitive and environmental), behavioral geography, and others, have collected evidence that familiarity is relevant to spatial cognition and navigation. Frequently, these research efforts relate familiarity to the general problem of spatial knowledge acquisition (see e.g. Montello^12^ for an early theoretical account suggesting that people, who are equally familiar with a spatial environment, may still differ with respect to the details of their mental representation thereof). Numerous studies have also indicated that familiarity impacts route instruction preference, route choice, and route memory. For example, in an early study, Peron and colleagues^18^ studied the types of objects (structural, movable, or fixed) recalled by students about to graduate (more familiar) versus first-year students (less familiar). The results indicated that unfamiliar students recalled about the same proportion of objects across category types, whereas the familiar group recalled different proportions. Lovelace and co-authors^19^ collected evidence that familiarity impacts the content and, hence, quality of route descriptions a person gives to characterize a route; depending on familiarity, different types of landmarks were used, such as on-route landmarks versus those located at decision points. Studying 45 first-year students and 45 fourth- or fifth-year students, Muffato et al.^20^ assessed the impact of familiarity and visuo-spatial factors. To this end, they used a jigsaw puzzle test, a mental rotation test, a sense of direction (SOD) and spatial representation questionnaire, a spatial anxiety scale, and an attitudes-toward-orientation scale as measures of visuo-spatial abilities; they assessed their impact on finding shortest routes and pointing accurately to landmarks on a university campus. They found that familiarity was a key factor in wayfinding performance but not in locating landmarks. Li and colleagues^21^ studied the impact of familiarity on verbalizing route instructions as well as sketching maps. They found that familiar participants with high spatial abilities overestimated distance considerably not only more than unfamiliar participants with high spatial abilities did but also more than the low spatial abilities group of participants regardless of their familiarity, whereas pointing accuracy got better with increasing familiarity. Familiar users sketched less distorted maps, and the orientation of those sketch maps differed between familiar and unfamiliar users.

### ML for ET and IMU data, and familiarity in the context of spatial cognition and wayfinding research

IMUs, which are worn on the head or body, have been extensively used in the domain of Human Activity Recognition (HAR; see Lara et al.^22^ for an overview), and a variety of Machine Learning (ML) techniques (e.g., Support Vector Machines (SVM), Random Forests, etc.) and deep learning methods have also been applied (see e.g. Eyobu et al.^23^) in this domain. Similarly, IMU sensors have been used to study/assist navigation behavior in-situ for some years (see Wang et al.^24^ for a very recent overview of pedestrian navigation activity recognition). For example, Jackermeier and Ludwig^25^ detect door transitions (when a person walks through a door to another room or space) from smartphone IMUs. By comparing different deep-learning architectures, Jackermeier and Ludwig provided evidence that reasonably accurate detection of transitions is sufficient to significantly increase positioning accuracy, solely based on Pedestrian Dead Reckoning (PDR). Aiming to increase positioning accuracy of PDR, Windau et al.^26^ studied ways to disentangle head movements and body movements with head-worn IMU sensors. Beyond mere improvement of PDR techniques to improve indoor positioning techniques, efforts to simultaneously classify types of motion (e.g., walking downstairs, running) and what a person is doing with a smartphone (e.g., holding the hand with the smartphone still) have also been made. Kasebzadeh and colleagues^27^ presented a neural-network architecture capable of jointly classifying motion type and smartphone position with 98% accuracy on the test set, a task that is important for wayfinding assistance systems that automatically change mode (e.g., audio to map-based) of route explanation.

To date, however, IMU sensors have not been used to measure environmental familiarity in-situ, i.e., based on Global Navigation Satellite System (GNSS) positioning data that are also collected while a person actually travels through an outdoor environment. This is in contrast to Eye Tracking (ET), which has been exploited frequently in spatial cognition research. For example, pupillary activity/gaze behavior has been successfully related to cognitive load based on task difficulty (see e.g. Kiefer et al.^28^) and time pressure (see e.g. Shojaeizadeh et al.^29^). Gaze behavior has also successfully been dynamically adapted to route instruction visualizations^30^.

While ML has frequently been applied to both ET and IMU sensor data, only a few studies have employed ML methods to classify environmental familiarity. Savage et al.^31^ used a Bayes Net classifier, exploiting data such as number of place visits or whether a person takes shortcuts to reach a place, in order to infer familiarity. In a Desktop-VR study, Gokl and colleagues^32^ collected data on a free-exploration task, running a binary classification task on field-of-view and joystick-based movement data that were used as a proxy for IMU data. Comparing random forests, logistic regression, gradient-boosted trees, and SVM, they found random forests to achieve the highest (albeit modest) accuracy of $65.70\%$. Recently, Liao et al.^33^ presented an in-situ study that used pupilometric, saccadic, and fixation-based gaze features to classify participants’ environmental familiarity. Participants were given a wayfinding task in which they needed to find their way on one familiar and one unfamiliar route (experiment 1) or two routes for each familiarity level (experiment 2). Using a random forests classifier, they found pupil diameter to be the most important classifying feature across routes and tasks. This, however, is a major weakness of this study per se, as the pupil diameter is not a meaningful feature in outdoor environments due to the lack of luminance control.

Our study design responds to several issues suggested by this literature review. First, based on the conceptualization and measurement of familiarity in the literature, we assess the familiarity of our participants in a multi-step procedure by asking them first to indicate familiar regions, familiar places, and familiar routes between pairs of these places. Second, based on prior empirical evidence about the impact of familiarity on aspects such as route knowledge, our study adopts a within-subject design that allows us to compare each person’s behavior on a familiar route to their behavior on an unfamiliar route. We hypothesize that particular eye/head/body movements of a person—behaviors—while traveling will reflect their state of spatial familiarity. Finally, we take a methodological step forward by simultaneously monitoring these behaviors using a combination of a head-mounted IMU sensor and a mobile eye-tracking device.

## Methods

### Study setup and procedure

In this section, we describe the setup and procedure of the study we conducted between June and October 2020. The study consisted of two parts. Using a custom-built website, participants first provided familiarity information (along with other data such as demographics, spatial abilities, and personality traits, none of which we use for the current paper). In the second part, participants were required to walk two routes, one in a region unfamiliar to them, and the other in a region familiar to them, using auditory, landmark-based route instructions (e.g., *Take a right turn after the Vinothek Rochus)*. During this walk, we record their locations, head and body movements, and gaze directions. The study was part of a larger data collection effort; hence, parts of the setup were first described in Golab et al.^34^; other parts of the data were used for analyses which were published earlier^2,35,36^. The consent form for participation and the study protocol were designed involving feedback by TU Wien’s Office for Responsible Research Practices in order to mitigate risks for participants as much as possible. Utmost care was taken to follow all applicable guidelines and recommendations for conducting this human subject study. All participants were older than 18 years of age and gave their written, informed consent to participate in this research as well as to the data privacy declaration of the research unit Geoinformation at TU Wien. 

#### Participants

**Fig. 1:**
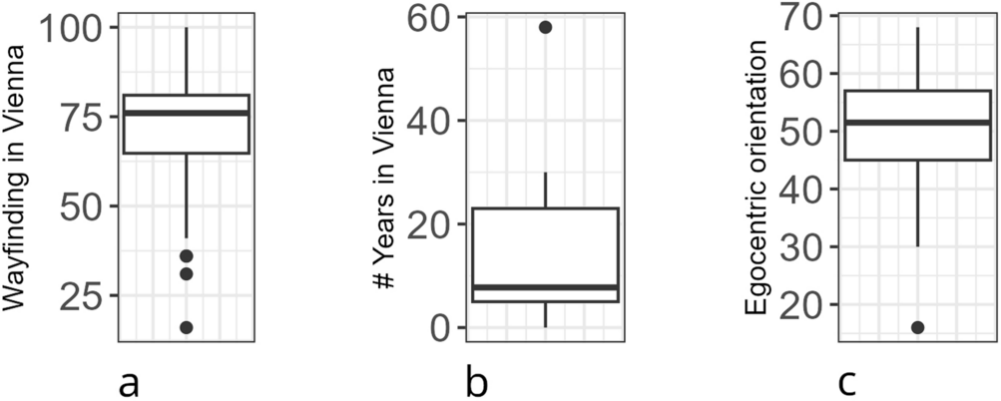
Boxplot (**a** left) shows the ratings how well participants find their way around in Vienna (analogue scale; 0: *not at all*, 100: *everywhere)*. Boxplot (**b** middle) depicts the number of years participants had lived in Vienna in total. Boxplot (**c** right) represents the scores of the global/egocentric sub-scale of the German-language FRS^37^; according to Münzer and colleagues^39^, this sub-scale measures a participant’s self-report sense of direction. Sub-figures produced using GNU R^40^ and the ggplot2^41^ package (v 3.4.1); throughout the entire paper we used GIMP to combine subfigures^42^ unless stated otherwise.

Overall, $N_{reg}=71$ people registered on our website and completed the demographics and related questionnaires. Of these, $N_{step12}=67$  finished steps 1 and 2 of the familiarity data collection (see below for an in-depth description of the collection of familiarity data). Step 3 was completed by only $N_{step3}=56$  participants and, of these, $N=52$ persons (female: 25, male: 27, $M_{age} = 26.2$ years, $MD_{age} = 24$ years) completed both in-situ parts (and we report all the further demographics for this group of people). Participants provided, first, demographic data etc. using LimeSurvey and were, subsequently, asked to provide their familiarity on a custom-built website (see below). In addition to age and gender, we provided a list of wayfinding aids (signs, maps, phones, passers-by, others) to participants and asked them to choose all of them which they use to navigate as pedestrians: $29\%$ of the participants report to use signs and their mobile phone, $23\%$ use maps in addition to these two and only $2\%$ report to use signs and/or phones in combination with salient landmarks. A third of the participants mentioned that they would also ask passers-by in addition to one or more of the other aids. We, furthermore, asked participants to self-report on how well they find their way around in Vienna (analogue scale; 0: *not at all*, 100: *everywhere*), how many years they have lived in total in Vienna, and to self-report on their spatial strategies using the German Fragebogen Raumliche Strategien (FRS)^37^, which comprises 19 questions all of which are rated on a 7-point Likert scale. As the left-most boxplot in Fig. 1 indicates, the majority of our participants report to find their way around in Vienna well; more than $75\%$ of all participants have lived in Vienna for five or more years (see Fig. 1b); Fig. 1c indicates that the majority of the participants considers themselves to have a good sense of direction: According to the norm data for the scale^38^, 56% of individuals younger than 30 years of age—a demographic that includes 48 out of 52 of the study participants—have a lower score.

#### Part I: collecting familiarity with regions, places, and routes

**Fig. 2:**
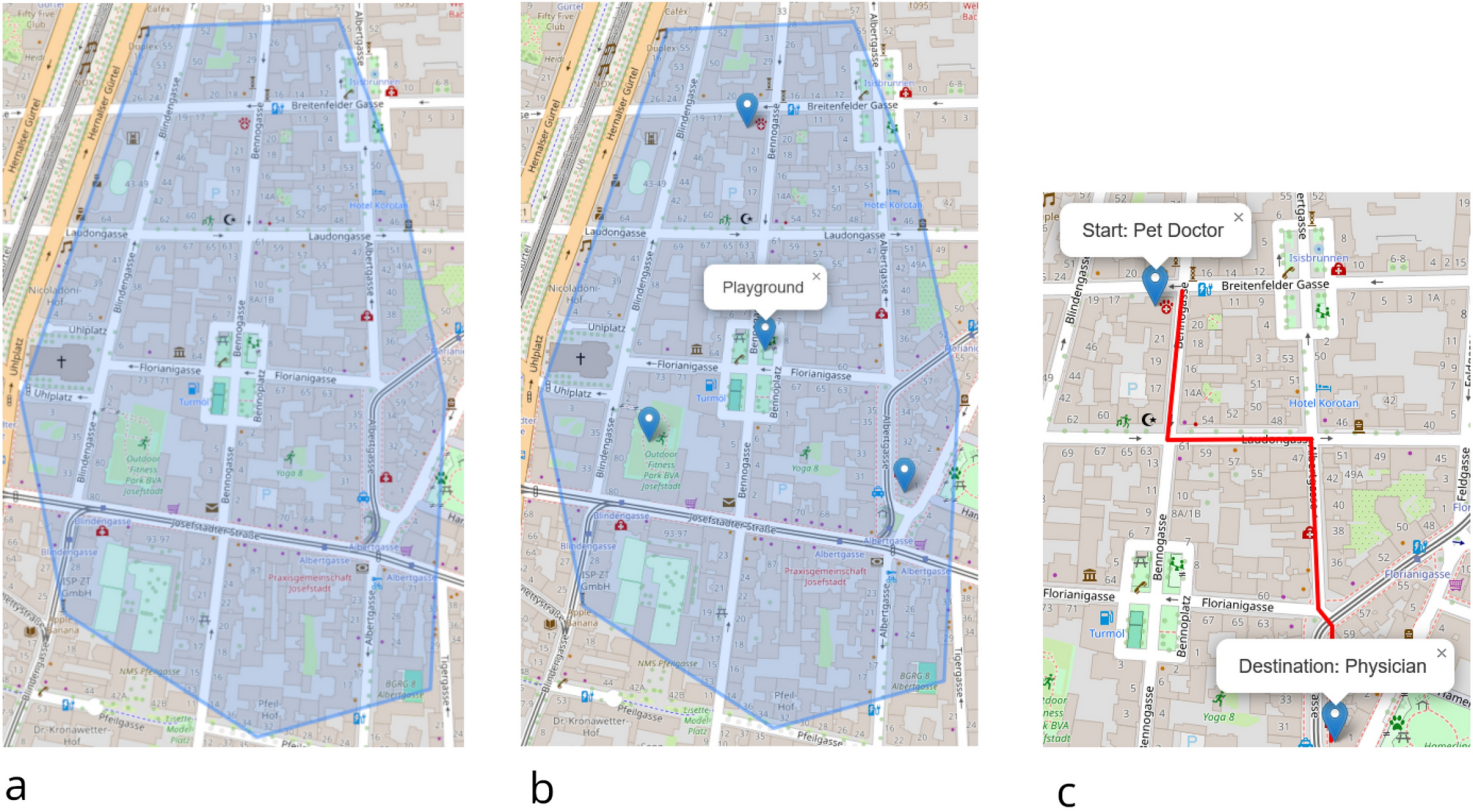
Illustration how we obtained familiar region polygons, places, and routes. *Step 1* (**a**, left): Participant marks a familiar region using a polygon (in blue). *Step 2* (**b**, middle): Markers are set on known places within these region polygons; participants add a label to each of these places (the graphic shows the label *Playground*). We chose two places randomly on the condition that their straight-line separation had to be 900 m–1.3 km. *Step 3* (**c**, right)): Participant draws route s/he would take between the selected places; both, start and destination are depicted using pins and a popup identifying them by name; top-left pin: *Start: Pet Doctor*; bottom-right pin: *Destination: Physician *. Basemaps are OpenStreetMap maps^44^; screenshots are taken from the actual user interface of the leaflet^43^-based web application.

Given the importance of the concept of familiarity for this study, we employed a multi-step procedure to collect information about participants’ familiarity with regions, places in those regions, and routes among these places (see Fig. 2 for an example picturing all three steps). While step 1 was carried out immediately after step 2, a time lag between steps 2 and 3 was unavoidable (see below).

- *Step 1: Familiarity with regions* Using a custom-built website based on the JavaScript library leaflet^43^, participants were presented with a map of Vienna. They were asked to draw a maximum of five region polygons on the map, each of which indicated a region in which they would know their way around without any navigational aids (maps and smartphone-based apps were named as examples of such assistance). To convey what we meant by such a region, an example from another Austrian city was presented to participants, but we emphasized that the region shown was only an example, i.e., the regions they would draw might differ in shape or size. As each region familiar to a particular participant would serve as an unfamiliar environment for another, participants were asked to refrain from drawing any regions within Vienna’s first district, as it is highly improbable that an individual has never visited this area. The user interface was deliberately kept as simple as possible; participants could view instructions at any time and tooltips were displayed in order to provide guidance. Participants outlined a total of $N_{reg}=184$ regions. The median area covered by the region polygons (see Table 1) was approximately $MD=2.1\, \hbox {km}^2$, and participants provided 2.8 regions on average (as explained above, they were instructed to provide no more than five regions; nevertheless, one person chose to provide six regions).

**Table 1:** Summary statistics for regions, places, and routes as provided by all participants doing this part of the study. Route lengths and straight-line distance between pairs of places are rounded to meters.

	Min	Max	Median	Mean	Stand. Dev.
Area of regions ($km^2$)	0.1	102.6	2.1	4.7	9.6
# regions per person	1	6	3	2.8	1.4
# places per person	2	65	20	21.8	14.2
Straight-line dist. of Points of Interest (POIs) pairs per participant (*m*)	6	15451	1007	1633	1872
Length of route (as sketched in *m*)	886	1453	1088	1115	142
- *Step 2: Familiarity with places* Within each of the submitted region polygons, participants were then asked to pinpoint 5–10 places (depending on polygon size) to which they were able to find their way without maps or smartphone-based assistance. They were explicitly made aware of examples such as local shops, subway stations, restaurants/bars, local schools, or visually salient buildings. Participants were also instructed to provide a name or label they would use to refer to these places when giving directions to others (examples included *Ikea* or *yellow building having red windows*). In order to enhance the descriptiveness of the procedure, participants were provided with the example of a picture for a region polygon located in a different city. Participants provided 1463 places in total, a mean of 21.8 places with an average pairwise straight-line distance of approximately 1.6 km. In Table 2, we provide an overview of the places reported, based on participants’ own descriptions. While approximately 12% referenced a building’s name or specific features of a building in the label, other frequent references include gastronomy services, such as restaurant, bar or cafe, supermarkets, or public transit stops.

**Table 2:** Classification of marked places based on the label provided by all participants doing this part of the study; relative frequencies are based on the set of labelled POIs which comprised 1449 cases.

Type of place description	%
Building	20.8
Restaurant/Bar/Cafe	10.1
Supermarket/Shop	13.5
Public transit stops	12.5
Educational institution	9.0
Square	5.0
Park	3.9
Church	4.6
Others	21.5
Marked places in total	1463 (labelled: 1449)
- *Step 3: Familiarity with routes* For each participant, we used all places indicated within a region polygon to find pairs of places with a straight-line distance of $900 \le d_s\le 1300$ m. We used this distance range in order to observe participant behavior for long enough walking time while keeping the overall walk to less than 20 minutes. A particular pair of places fulfilling the distance requirement was randomly chosen. One of the two places was randomly selected as a starting point, labeled *start* on the map image; the other place was labeled *destination*. These calculations were done off-line, resulting in a median time lag between steps 2 and 3 of 9.6 days. Participants were reminded about step 3 by email and received a personalized link to perform the following task: Each participant was asked to draw a line on the map to represent the route s/he would most likely take between the two places. We explicitly told participants to draw a route along pedestrian pathways or streets but not going through buildings or private property. Again, we provided an example picture to participants of what they were required to do. In total, participants provided $N_{step3}=56$ routes (i.e., we saw a dropout of 11 participants). Table 1 contains summary statistics of the lengths of the sketched routes which were, on average, approximately 1100*m* long.

#### Part II: in-situ wayfinding study

##### Materials

 For each of the routes we collected in Part I, route instructions were designed for segments bounded by turning points, following the concept of *spatial chunking* described by Klippel and colleagues^45^. Each route instruction was formulated based on the algorithm described by Roussel and Zipf^46^, within which POI data points drawn from OpenStreetMap (OSM) around a decision point are considered for reference as landmarks in the route instruction. The objects represented by the POIs are assigned a suitability metric based on their salience, uniqueness, advance visibility, position relative to the decision point, and direction of travel. The object with the highest suitability metric value was included in the instruction. Due to missing POI data in the OSM database, the algorithm was only able to suggest a landmark for 77% of the turning points of the routes. We checked all route instructions in the field in order to avoid problems such as overestimated object salience or ambiguous reference. For 37% of the turning points, the landmark suggested by the algorithm was observed to be adequate. The remaining 63% of the landmarks, however, had to be altered, primarily due to their potential ambiguity as instructions or their poor salience (further details on this can be found in Golab et al.^34^). The formulated route instructions were synthesized using Google cloud Text-to-Speech Engine^47^. They were auditorily presented to participants through earphones.

**Fig. 3:**
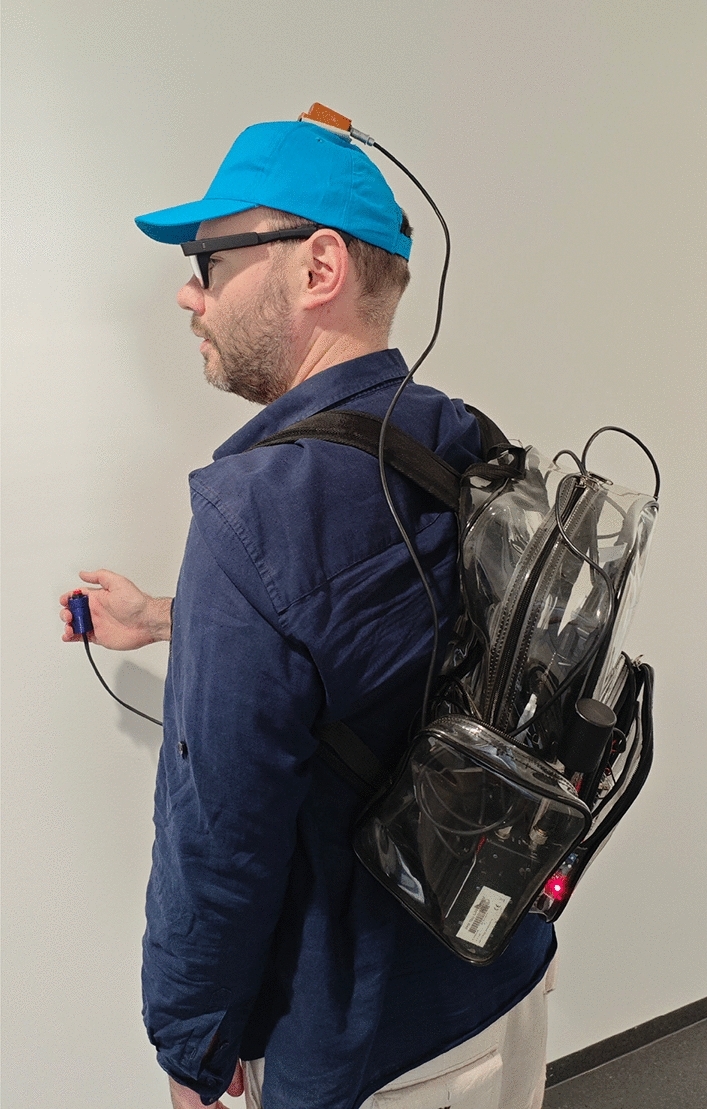
A person (informed consent for use of image granted in writing) showing the equipment which participants wore during the study. The head-mounted IMU was attached to a baseball cap which shaded the infra-red cameras of the ET glasses. The clicker (right hand) was used to light a red LED, indicating a route instruction request. The high-precision GNSS receiver was placed in the outer bag of the backpack.

During the study (see Fig. 3), the participants’ eye movement data was collected using the PupilLabs Invisible device and head movement data by mounting the Xsens MTi-300 IMU on the top of a cap worn by the participant the head, oriented approximately with a person’s forward-facing view. Furthermore, locational data was collected using the PPM 10-xx38 GNSS receiver. Participants also carried a custom-build clicking device to request route instructions (see Section “Procedure”).

##### Procedure

 During the study, each participant walked two routes, one familiar and one unfamiliar. The familiar route was provided by the participant during Part I. The unfamiliar route was chosen randomly from the pool of routes provided by other participants, within a region not familiar to the participant. The order of which of the two routes participants walked first, was randomized. The median number of days between the two trials per participant was 0 (i.e., participants did both trials on the same day), and for 75% of all participants, the two trials were not more than 8 days apart. Due to the COVID-19 pandemic, however, the time lag between step 3 and the first trial was much longer than originally intended (median: 68 days).

Before walking each of the routes, the experimenter did a time sync procedure for the sensors by asking the participant to look at the mobile phones collecting the ET data, the GNSS data, the phone from which the route instructions were played, and the laptop used to collect the IMU data (see Section “Data preparation and feature engineering” on how the resulting ET video data was used). Subsequently, the researcher explained the route instructions by means of an example. Participants were equipped with wireless earphones and were told to follow the auditory instructions carefully. In the case of familiar routes, it was explained to participants that the route might differ from the one they had provided online, and participants were reminded of the destination to increase the chances they would remember the route. Participants were accompanied by the experimenter, who walked behind the participant at a few meters distance. Throughout the study, participants were free to request route instructions as often as they wanted. They issued a request using a custom-built clicking device that lit up a red LED light, alerting the researcher to replay the route instruction via Bluetooth (the researcher made sure to play the route instruction to participants immediately after the LED light was lit up). In $68.8\%$ of cases, participants chose to request a route instruction only once, whereas in $31.2\%$ they issued at least a second request (see Alinaghi et al., p. 1:6^48^).

### Data analysis

#### Description of available data

Based on the data collection procedure described above, the available raw data comprises:

- **Location data** Collected using a PPM 10xx-38 GNSS receiver, providing a recording frequency of 1*Hz*.
- **Mobile eye-tracking data** Recorded using a Pupil Labs Invisible mobile eye-tracking device; the collected data comprised both, raw x, y gaze positions and a first-person, scene video recorded at 30 frames per second, providing a visual record of the surrounding environment. The raw x, y gaze positions were post-processed to a frequency of 200 Hz in the Pupil Labs cloud.
- **Head-mounted IMU data** Using an Xsens MTi-300 providing six degrees-of-freedom sensor data to sense acceleration, pitch, roll, and yaw. It is important to note that the position and alignment of the device on the heads of participants is inevitably inconsistent between trials due to the in-situ nature of the study.

#### Data segmentation

**Fig. 4:**
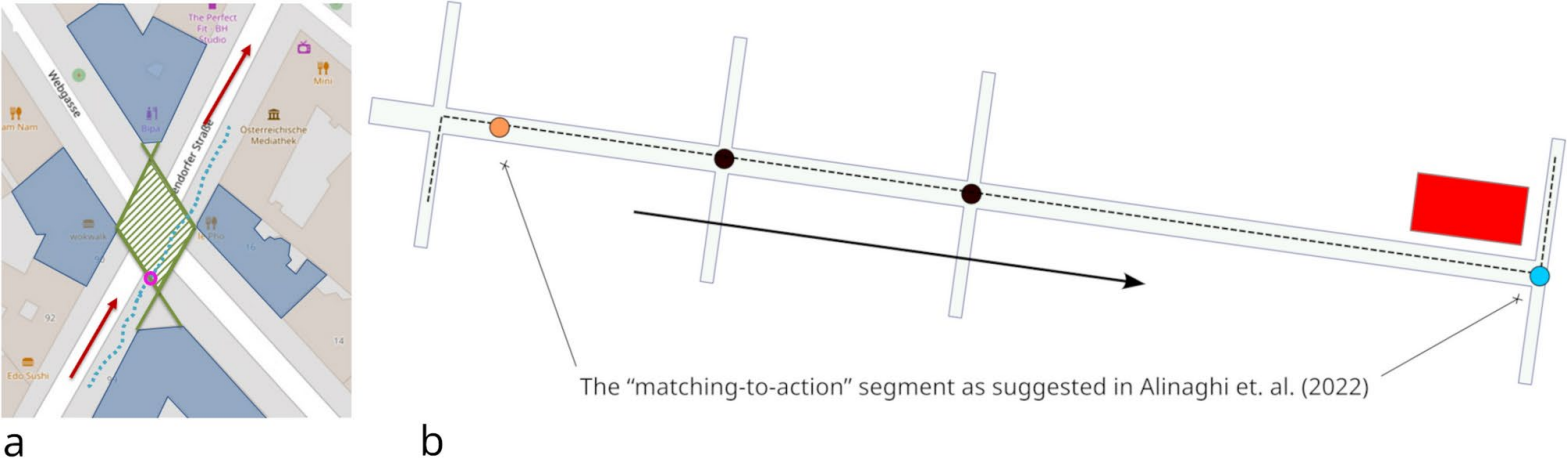
Visual explanation of the data segmentation used. (**a**): Explanation of how the decision area is determined based on the building footprints located at an intersection (reprinted from^35^; blue polygons: building footprints; hatched green polygon: decision area; dashed blue line: GPS trace; pink circle: decision point; red arrows: direction of travel figure (**b**): First the “matching-to-action” part(s) of the route are extracted, which range from the location at which a route instruction was requested [orange dot] to the junction it refers to [blue dot; example instruction: *Turn left after the red house.*]). Then, a non-overlapping 3-s sliding window is applied to these parts; black arrow: travel direction; black dots: non-turn junctions; dashed black line: route. Basemap for figure (**a**): OpenStreetMap^44^; figure b created using Inkscape^49^.

Following the approach of Alinaghi et al.^2^, who segmented a route based on what they termed the “matching-to-action” phase, we applied a similar segmentation method for our ML experiments. Since the provided instructions were specific to turns (e.g., “Turn left at the [Landmark]”), we focused on the segments of the route that represent the decision-making and wayfinding behavior triggered by the instructions. These segments span from the moment an instruction is received by the wayfinder to the moment they reach the specified junction (see Fig. 4, where the route instruction request is shown as an orange dot and the junction it corresponds to as a blue dot). We segmented this part of the route using a non-overlapping sliding window with a fixed window size of three seconds, as recommended by Alinaghi and colleagues^2^.

#### Data preparation and feature engineering

##### Synchronizing IMU data without timestamps

**Fig. 5:**
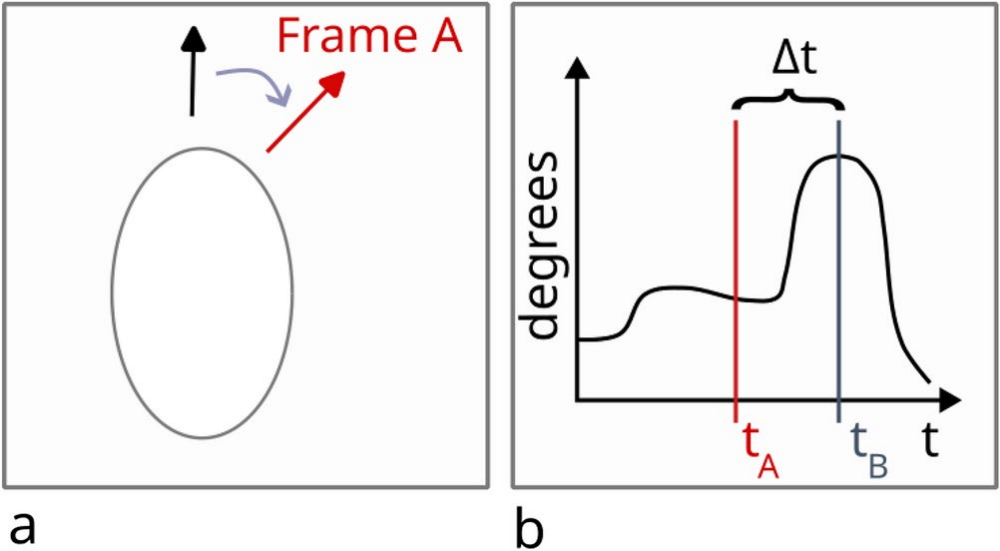
Synchronization of IMU data without timestamp: (**a**) Extraction of the time frame in eye-tracking videos during which an extreme head movement is made, shown from a top view of a participant’s head; the black arrow indicates the walking direction, the red arrow indicates the changed X direction resulting from the quick head movement to the right; (**b**) Time lag between an IMU time with low accuracy and the true acquisition time is determined; the shown curve represents the time series of acceleration in x-direction; $t_B$ denotes the yaw peak. Figure created using Inkscape^49^.

 In 31% of cases, the timestamps of the IMU data were not recorded due to a data sampling issue during the outdoor study. Hence, for these cases, only the creation timestamp of the log file with 1 sec precision is known. We synchronised these parts of the IMU data to the locational log files to sub-second precision through the following approximation procedure (see Fig. 5). For each of the affected trials, we manually searched for three to four cases in which the first-person, scene video revealed a rapid horizontal head movement by the participant. This movement corresponds with extrema in the IMU’s yaw measurements, and identifying several of these occasions increases the robustness of our approximation. In order to increase the resolution of the timestamp to less than one second, we evaluated the extremum within the time frame of one second before and after the timing of the video frame in which the rapid movement occurred. The time lag $\Delta t$ between the extreme head movement at the resolution of one second, $t_A$, and the detected extreme point in yaw measurements, $t_B$, is then calculated.

##### Synchronizing GNSS, IMU, and ET data

The time of all sensors involved was synchronized to GNSS time using the following procedure:

1. Assign frame number 0 of the ET video to the start timestamp contained in the recording’s metadata file provided by the PupilLabs mobile ET device. This is, in fact, the timestamp in which an eye-tracking recording was started.
2. Using a Java application, we displayed the timestamp of the laptop’s system clock, to which the GNSS and the IMU sensors were attached. This event was recorded by the first-person, scene video, and we found the corresponding frame number of the ET video which shows the timestamp.
3. Determine the frames per second of the first-person, scene video, using opencv version 4.5.2.54.
4. Using the frame numbers and the number of frames per second, find the GNSS time for the IMU and the ET data.

#### Data filtering

As each participant of the $N=52$ participants had to walk in a familiar and an unfamiliar environment, we collected data from $N=104$ walks. We applied a highly conservative case-wise deletion procedure for those recordings for which (1) a malfunctioning occurred of any of the sensors (GNSS, IMU, ET, or clicker device) used to collect route instruction requests (e.g., the IMU recording would stopped early or the file containing the route instruction requests was not written to the file system; this occurred quite frequently, accounting for 33 cases); (2) rarely (5 cases) time synchronisation issues occurred (stemming, e.g., from the fact that one of the systems used to collect the sensor data would crash); and (3) very occasionally (3 cases) human error was present in those files, representing the location of decision points. We, therefore, excluded an overall number of 41 recordings, resulting in an overall number of $N_{ov}=63$ recordings remaining for further analysis. For the purpose of further data cleaning, all first-person, scene videos collected with the mobile eye tracker were screened manually using PupilPlayer v3.5.1. Based on the video play time (precision in milliseconds), we identified the beginning and end of each trial as well as any interaction between participant and researcher during the trial which would interrupt user behavior. The majority of these cases occurred due to inevitable checks for the malfunctioning of some parts of the equipment, but other causes also happened, such as interactions between participants and people they would know in the streets. By analogy with the procedure described in steps three and four above (see Section “Synchronizing GNSS, IMU, and ET data”), these points in time were synchronized to GNSS time and, subsequently, used to remove any data within these time frames from the GNSS, IMU, and gaze position files. The cleaned IMU data were further filtered for anomalies, which occurred due to the adverse study conditions, such as when participants would move the cap on their head due to windy conditions (participants were, of course, explicitly instructed not to touch the cap). In order to avoid a bias induced in free acceleration and further IMU data due to these issues, we cleaned the data by exploiting a change-point detection method (see Aminikhanghahi and Cook^50^ for an overview of available methods). Following the approach taken by Bouwman and colleagues^51^ for step detection, we first applied a low-pass filter to the free acceleration data along the x ($fAcc_x$), y ($fAcc_y$), and z ($fAcc_z$) axes; we then continued detecting change points using a single spectrum transformation as provided by Takehiro Suzuki’s implementation available at the following URL https://github.com/statefb/singular-spectrum-transformation (last accessed 2024-08-19). We classified the entire signal of a trial into *desired* and undesired classes based on a $10 m/s^2$ threshold, which we identified in a pilot study during which we simulated both desired and undesired behaviors. We excluded all signal parts classified as undesired from the IMU time series; as the eye movements we recorded may also have been affected by these issues, we also dropped them from the eye-movement time series as appropriate.

##### Mapping GNSS data to the street network

For each trial, we manually checked the collected GNSS data to see whether it reflected a participant’s actual trajectory. We distinguish two different cases:

- **Factually wrong**. In 63% of cases, the GNSS trace crossed building footprints or indicated changes of street sides by participants when this was, in fact, not the case. These GNSS data were projected onto a manually drawn linestring which indicated the true route participants walked, rather than using unmatched GNSS traces that were inaccurate.
- **Sudden inaccuracies**. For the remaining 37% of cases, sudden inaccuracies occurred (e.g., due to a temporary loss of the real-time kinematic ground correction of the signal). We smoothed each of these GNSS traces by calculating a weighted average of the coordinates of four points, two measured right before the timestamp of the suddenly inaccurate location and two right after.

#### Downsampling IMU data

The Xsens MTi300 sensor records data at different frequencies with nanosecond precision. Inertial data (e.g., acceleration, free acceleration, rate of turn, etc.) are recorded at a frequency of 400 Hertz (Hz), whereas orientation data (e.g., yaw) are recorded at a frequency of 100 Hz. The sensor also provides high resolution (HR) data, including acceleration HR and rate of turn HR, which are recorded at 1000 Hz. Recent evidence suggests that it is useful to downscale IMU data when applying ML data analysis methods, as this will increase the efficiency of the model (see Kim et al.^52^). Moreover, the results presented by Kahn and colleagues^53^ demonstrate that it is possible to reduce the sampling frequency of accelerometers without losing accuracy in detecting a wide range of activities. So far, however, we do not know the highest possible frequency of movements because we lack empirical IMU data on the head movements of wayfinders in urban environments. We, therefore, chose to downsample the data to the lowest frequency of the important measures we need for our human-activity detection analysis (see Khan et al. for this advice^53^): Arguably, rotational measures (roll, pitch, yaw) are more important than translational features when it comes to head motion measurement. As these measures were recorded at a frequency of 100 Hz, we downsampled all other measures based on the sampling timestamp matched to the recorded timestamp of the rotation data.

### Feature extraction

After preprocessing the recordings, we developed features for gaze and IMU data. For gaze data, we used the common fixation-based and saccadic features proposed by Alinaghi and colleagues^35^ for the purpose of activity recognition in wayfinding For IMU data, we employed a set of features used in signal analysis in various fields, ranging from animal motion-behavior studies to music genre classification.

#### Engineering features for eye-tracking data

Using the gaze positions tracked with the mobile eye-tracking glasses, we calculated several fixation-based and saccadic features (see Table 3). Since a 200 Hz gaze-recording frequency does not allow for velocity-based gaze event detection, we utilized the commonly used dispersion based Identification by Dispersion-Threshold (IDT) algorithm^54^ to detect fixations (gaze-dispersion threshold: 0.02; we use the normalised pixel distance on the frame as the unit of measurement because in mobile eye-tracking, the distance to the stimuli is not known, and measurements in degrees are therefore meaningless; time threshold: 100 ms)^1,30^. Saccadic features, however, are not calculated solely based on this set of fixations due to the presence of head-movements during recording. It is known that the analysis of saccadic eye movements is more complex in mobile ET as compared to stationary ET, as the stimuli and observer move dynamically. In particular, saccade amplitude and velocity are significantly impacted by head movements^55^. At the same time, saccadic features are important features for gaze-based activity recognition^35^. We, therefore, used the algorithm proposed by Alinaghi and Giannopoulos^56^ to calculate features related to saccade length, including the mean/minimum/maximum/variance/skewness of saccade amplitude and the g-l ratio, i.e., the ratio between long and short saccades. This method is based on processing the video frames of the scene with an image stitching algorithm. Image stitching algorithms generally estimate the rotation, scale, and transition between each image and a reference image, in the form of a homography matrix. When successive images including a common saccade are merged, the rotation elements of the homography matrix can be viewed as an estimate of the head movements during the saccade. These elements translate the roll, pitch, and yaw caused by the head movements. The effects of these rotations are applied to the corresponding fixation points of the saccade to project them to the correct coordinates. Thus, we used this algorithm to calculate the refined saccadic features considering head movements.

**Table 3:** Gaze-based features extracted for familiarity classification. For each row, the rightmost column indicates the final number of features we obtained by applying the statistical measures presented in the first column to the gaze features represented in the second column. For instance, the first row of saccade-based features encodes eight features, namely, mean/min/max/var for both saccade amplitude and saccade duration.

Fixation-based features		
Mean, min, max, var	Duration, dispersion, dispersion X, dispersion Y	16
Frequency	–	1
Saccade-based features		
Mean, min, max, var	Amplitude, duration	8
Skewness	Amplitude	1
Frequency	–	1
g-l ratio (the ratio between long and short saccades)	Amplitude	1

#### Engineering features for IMU data

HAR research focuses mainly on the recognition of physical activities that involve certain types of movements (see Lara et al.^22^ for a review). The closest work in the field of HAR to our problem is the work by Vanrell and colleagues^57^. They tested a variety of feature combinations for human activity detection and employed cepstral coefficients, time domain measurements, the fundamental period, and the use of magnitude of acceleration versus x,y,z acceleration components on the widely-used dataset presented by Xue and Jin^58^.

Given the sparseness of available studies within the HAR domain, we also considered literature concerning engineering methods from other fields that analyze signals similar to ours. We particularly focused on research efforts about subtle, almost visually indistinguishable patterns, as we expect familiarity also not to cause differences visible to the naked eye. Both animal behavior studies, and studies on music analysis and genre classification, seemed to be relevant domains (discussed below).

Based on this approach, we used the following IMU features in this paper. We use relative values where appropriate due to the fact that it is impossible to mount the IMU at the exact same position of each participant’s head. The entire list of 99 features from the IMU data can be found as Supplementary Information online (see below):

- Basic statistical features on raw data (minimum/maximum/average/variance of relative acceleration along x, y, and z axes; minimum/maximum/average/variance of relative roll, pitch, and yaw; frequency change in yaw; point-to-point amplitude for acceleration; and free acceleration along the x, y, and z axes).
- Several of the measures suggested by Vanrell et al.^57^:
  - **Cepstral coefficients**. The Cepstral coefficients are the coefficients of the cepstrum, which is, in turn, obtained by calculating the inverse Fourier transform (IFT) of the logarithm of the estimated signal spectrum (see e.g. Norton et al.^59^). These features have been widely used in audio signal processing for speech recognition^57^ and music information retrieval^60^. The reason is that these coefficients are compact representations of the original signal in a dimension where the noise-immune patterns are well represented. The cepstral coefficients were computed from the acceleration signal at a sampling rate of 100 Hz (equal to our data frequency), and the first 50 coefficients, emphasizing the higher-order coefficients, were considered by analogy to Vanrell et al.^57^.
  - **Time-domain measures (TDM)**. These are the standard deviation, energy (the indefinite integral of the squared magnitude of the acceleration signal), and minimum/maximum/peak-to-peak amplitude of the acceleration signal.
  - **Fundamental period (FP)**. This is the smallest time difference between two subsequent amplitude peaks of the acceleration signal; the fundamental period contains information about the velocity with which a person moves through space.
- Fogarty and colleagues^61^ use the following features to distinguish the less obvious differences in movement patterns of sheep carrying out activities such as grazing, lying down, standing, and walking. As we expect subtle differences between familiar and unfamiliar people, we deemed these acceleration-based features worthwhile to consider; all are calculated within each time window:
  - **Movement intensity (MI)**. These measures are based on the square root of the sum of the squared acceleration along all three axes.
  - **Signal magnitude area (SMA)**. This is the area under the acceleration signals of all three axes divided by the duration of the time window.
  - **Energy**. This is the sum of the squared acceleration for each axis, normalized by the number of records within the time window.
  - **Entropy**. By analogy to Shannon’s information entropy, this is a measure of the uniformity of a signal; the more irregular the signal is within a time window, the higher the signal’s entropy is.
  - **Movement variation (MV)**. This measures the acceleration variability based on the sum of the absolute change in acceleration along all three axes, between all pairs of subsequent data points sampled, normalized by the number of data points obtained within the time window.
- Audio signals are sometimes so visually similar that music analysts need to extract more complex features from them in order to detect differences between different types of music. Again, we anticipate that familiar and unfamiliar participants’ IMU signals may be this similar too. Following Bahuleyon’s work^62^, we consequently employ the following features:
  - **Zero-crossing rate**. This is a weighted measure of the number of times the signal changes sign within a given time frame. Hence, it indicates the smoothness of the signal.
  - **Spectral centroid**. This measure is used in digital signal processing to characterize a spectrum. It indicates where the center of mass of the spectrum is located.
  - **Spectral rolloff**. This is the frequency below which a specified percentage of the total spectral energy lies (e.g., 85%).
  - **Chroma frequencies**. Chroma features are representations for music audio in which the entire spectrum is projected onto 12 bins representing the 12 distinct semitones (or chroma) of the musical octave. In contrast to reporting the 12 (or more) frequencies, which is common in music analysis, we have used the mean of the twelve bins for the first timeframe as a feature (in general, the mean of the 12 frequencies represents information about the overall pitch energy).

## Results

We briefly describe the classification technique we used and then report the results of our ML experiments based on gaze features, IMU features, and their combination. The results reveal the superior importance and decisiveness of IMU features over gaze features.

The predictive models we used to classify behaviors were built using the powerful gradient-boosting technique (see e.g. Hastie al.^63^, chap. 10), which is based on the idea that several so-called *weak learners* (i.e., models which do not perform well when used alone, such as single trees) are made stronger by having each tree try to minimize the error rate of the previous trees. Gradient Boosted Trees are a particularly promising application of this technique, allowing the modelling of complex relationships. We used the XGBoost^64^ implementation in this work, as it is scalable and highly efficient; in addition to that, we achieved very reasonable results with this model in previous work^2,35,36^. The data was filtered such that any data points that deviated by more than three standard deviations from the mean value were eliminated. We chose two approaches for training and testing the models: First, a common 80/20 split for training and test with a 10-fold cross validation for hyperparameter tuning (see Table 4 for the list of parameters we tested). Second, in order to ensure there is no data leakage and to increase the generalizability of our results, we kept the data of five, randomly selected participants per condition for testing, i.e. we used this data neither for training nor validation. We refer to this method as leave-five-out-for-testing (L5O4T) throughout the paper and have chosen to select datasets per condition (rather than five participants and leave all the person’s data across conditions out for training) because we hypothesize that the degree of familiarity impacts the behavior. This accounts for roughly 20% of the data, making the size comparable to the 80/20 split. Again, we used a 10-fold cross-validation for hyperparameter tuning. To increase the generalizability of the results in the L5O4T method, we repeated the training/testing experiments ten times, each time with five different randomly selected participants per condition. We report the average, best, and worst case results in order to double-check for any influential individual patterns. We used the trained models for binary classification of the data, and we plotted the *logloss* to examine potential under- or over-fitting (we used negative log-likelihood as a scoring function, see Fig. 8). Finally, we used the SHapley Additive exPlanations (SHAP) method to calculate the feature importance^65^, helping us to interpret our results. When interpreting the SHAP-based feature importance, it is important to keep in mind that this method takes multicollinearity between features into account^66^.

As visual search is typically essential for orientation by people not visually impaired, we first examined gaze fixations and saccadic events (see section above). In contrast to our expectations, however, we achieved weak results for a binary classification problem: The range of the accuracy values is $53.4$–$76.8\%$ (L5O4T worst vs. L5O4T best case), and are almost identical for the average L5O4T case ($67.6\%$) and the 80/20 ($66.2\%$) splits.

**Fig. 6:**
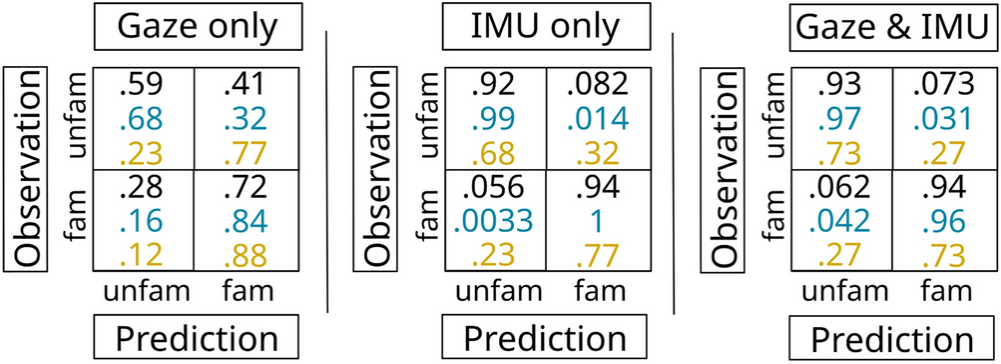
Confusion Matrices for the ML experiments using a 80/20 training-test split (black), and the best (blue) and worst (yellow) case result of the L5O4T training-test split. Numerical values calculated using Python 3^67^; figure created using Inkscape^49^.

We then examined prediction using exclusively IMU features for classification. Except for the worst-case based on L5O4T ($72.8\%$) split, this model achieved a much higher classification accuracy of $93.9\%$for the 80/20 split, $99.1\%$ for the best-case L5O4T, and $89.9\%$ for the average-case L5O4T. Finally, we used the gaze and IMU features together as input. The achieved overall accuracy, however, was actually lower than when using IMU features only across validation strategies except for the worst-case L5O4T, which remained unchanged. In case of the 80/20 split we see a slight drop in accuracy ($0.6\%$), whereas for the best and average case L5O4T, the drop in accuracy when using both, gaze and IMU features is substantial (approx. 3% for the best case and $4\%$ for the average case).

**Table 4:** Overview of the performance of each of the three models (gaze only, IMU only, and the combination of both feature sets) across different training-test splits of the data, including the resulting hyper parameters.

TTS	Data	#feat	Scor	#iter	#spl	Acc (%)	Sub	#est	min_chw	max_d	l_rate	$\gamma$	cs_bt
80/2	gaze	26	n_l_l	1000	10	$66.2$	0.4	700	0.5	6	0.01	1.0	1.0
L_b	gaze	26	n_l_l	1000	10	$76.8$	0.5	500	0.5	6	0.01	0.6	1.0
L_a	gaze	26	n_l_l	1000	10	$67.6$	–	–	–	–	–	–	–
L_w	gaze	26	n_l_l	1000	10	$53.4$	0.1	500	0.7	3	0.001	1.0	0.7
80/2	imu	46	n_l_l	1000	10	$93.9$	0.5	1000	0.6	10	0.05	0.7	0.8
L_b	imu	46	n_l_l	1000	10	$99.1$	0.5	100	0.5	10	0.1	1.0	0.6
L_a	imu	46	n_l_l	1000	10	$89.9$	–	–	–	–	–	–	–
L_w	imu	46	n_l_l	1000	10	$72.8$	0.5	100	0.5	10	0.1	1.0	0.6
80/2	g + i	72	n_l_l	1000	10	$93.3$	0.5	1000	0.6	10	0.05	0.7	0.8
L_b	g + i	72	n_l_l	1000	10	$96.3$	0.5	500	0.5	6	0.01	0.6	1.0
L_a	g + i	72	n_l_l	1000	10	$85.9$	–	–	–	–	–	–	–
L_w	g + i	72	n_l_l	1000	10	$72.7$	0.5	500	0.5	6	0.01	0.6	1.0

Columns are: *TTS:* train-test split (*80/20:* 80/20 split; *L_b:* L5O4T best case; *L_a:* L5O4T average; *L_a:* L5O4T worst case); *#feat* number of features; *scor* scoring (*n_l_l* means negative log-likelihood); *#iter* number of iterations; *#spl* number of splits; *Acc* accuracy; *sub* subsample; *#est* number of estimators; *min_chw* minimum child weight; *max_d* max depth; *l_rate* learning rate; $\gamma$ Gamma; *cs_bt* column sample by tree. In column *Data*, $g+i$ denotes combined gaze and IMU features.

Table 4 provides an overview of the number of features used, number of splits, number of iterations, scoring, and the XGBoost parameters yielding the highest accuracy for each of the three models and both training-test split approaches. Figure 6 gives the confusion matrices for the 80/20 training-test split in black, the best case based on the L5O4T split in blue, and the worst case in yellow. Across validation techniques, the confusion matrices indicate that confusing unfamiliar cases with familiar cases is more common than when using either only gaze or only IMU data.

**Fig. 7:**
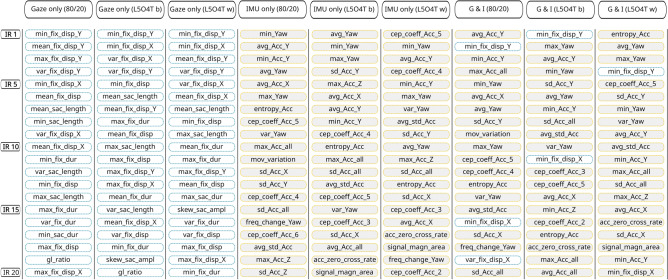
The SHAP-based feature importance for all three models for both validation strategies. For the L5O4T training-test split we present the best (b) and worst (w) cases. *IR* means *importance rank*. Feature acronyms: *fix* $\rightarrow$ *fixation*; *sac* $\rightarrow$ *saccade*; *skew* $\rightarrow$ *skewness*; *ampl* $\rightarrow$ *amplitude*; *dur* $\rightarrow$ *duration*; *magn* $\rightarrow$ *magnitude*; *acc* $\rightarrow$ *acceleration*; *cep* $\rightarrow$ *cepstral*; *cross* $\rightarrow$*crossing*; *mov* $\rightarrow$ *movement*; see supplementary material for the full list of IMU features and Table 3 for the gaze features. The figure is based on the numerical results calculated using Python 3^67^ and the XGBoost package^64^.

**Fig. 8:**
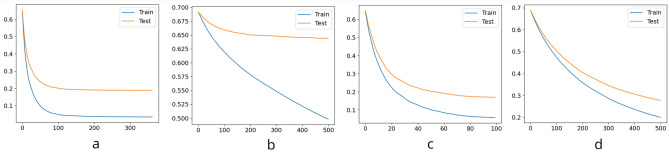
The log-loss functions (train in blue, test in orange) for the best performing models; (**a**): 80/20 split | IMU only; (**b**): L5O4T | gaze only; (**c**): L5O4T | IMU only; (**d**): L5O4T | gaze and IMU. The y-axis shows the log-loss, the x-axis represents the number of trees.

In order to gain insight into the importance that different features have for classifying a user’s familiarity, we computed the SHAP values and report the rank of the 20 features which have the highest impact on the model output magnitude (see Fig. 7) across models and validation strategies.

Using exclusively gaze data, features related to fixation dispersion are most important and also predominant across different train-test split strategies, as 7–8 out of the 10 highest ranked are fixation-dispersion related; overall, only 4-6 of the 20 most important features are saccadic features. When classifying familiar and unfamiliar wayfinders based on head-mounted IMU data, we see a prevalence of yaw and acceleration-related features. For the best-case L5O4T the average, min, and max yaw are the three most important features; for the 80/20 split case min yaw is most important and average and max yaw are also among the six most important features; for the worst-case L5O4T, however, a single yaw-related feature is ranked among the top-five. Combining gaze and IMU features, only three features out of 20 are gaze related in case of the cross-validation based on an 80/20 split, and two out of 20 features for the L5O4T-based experiments. Irrespective of the method by which training and test sets are created, minimum fixation dispersion along the y-axis is the only gaze-related feature among the top ten (ranked second for, first and fifth).

## Discussion

Conducting ML experiments with three different sets of features (gaze only, IMU only, and gaze + IMU), our results clearly indicate the superior classification performance of using exclusively IMU features. This result is contrary to our expectations, which were that the combination of gaze and imu features would perform best. In addition to that, the particularly weak performance when using exclusively gaze features was unexpected too. As a consequence, we first discuss the superior performance of IMU features, then discuss the weak performance of gaze features, and, finally, touch on the reduced accuracy achieved for the combined feature set.

We achieved a high accuracy of $89.9\%$ based on the average of the experiments using a L5O4T training-test split. This is a very reasonable level of accuracy as the testing is done on completely unseen data in this case (as in opposition to the 80/20-split experiment which has parts of the data of each participant, resulting, thus, in a higher accuracy). This level of accuracy is especially impressive when compared to other human-activity recognition studies. For example, Gokl and colleagues^32^ reported only a modest accuracy of $65.7\%$ for their binary classification task of familiarity based on joystick motion in a Desktop-VR setting. The accuracy we achieved is comparable to the one by Kasebzadeh et al.^27^, but that was for classifying motion type and smartphone position. Having said this, we see considerable variation in accuracy between the best-case ($99.1\%$) and the worst-case ($72.8\%$) L5O4T-based experiments. Apparently, there are very large individual, behavioral differences, i.e. there are people who show movements which decisively help to distinguish familiar from unfamiliar participants; on the other hand, there are other people, who do not have these. Across all participants, however, the classification works very well. However, due to the fact that we used only a single IMU sensor, we cannot easily disentangle the head rotation acceleration from the acceleration resulting, e.g., from walking patterns related to gait (see also Windau and Itti^26^). As we see in the importance ranks of SHAP values, yaw and acceleration along the y-axis are the most important features. Head movement in the form of acceleration and rotation along the y-axis, indicating orientation behavior while walking on the street, is informative: Familiar and unfamiliar wayfinders have different visual search behaviors, and this difference is reflected in the yaw of the head and the acceleration along the y-axis of the right-handed IMU coordinate system. However, for the best-case L5O4T the maximum acceleration along the z axis is of considerable importance. This points, again, towards a difference in walking behavior. Similar to the case of yaw and acceleration along the y-axis, however, it is very challenging to disentangle the impact of gait from full-body or upper-body rotation due to the use of a single, head-mounted IMU.

Using gaze features only, the achieved classification accuracy was unexpectedly weak. Even more strikingly, the confusion matrix (see Fig. 6, left) suggests that while classifying familiar people as familiar worked reasonably well, the classification of unfamiliar people as unfamiliar was was very weak even for the best-case run using L5O4T. In fact, this result is generally in line with the findings by Liao and colleagues^33^, who also observed a difference in precision between familiarity levels when using what they call basic statistical features. Nonetheless, we find this match of results surprising for two reasons: (1) In contrast to Liao et al.^33^, we take head movements into account when calculating saccadic features (see section), and (2) unlike Liao et al.^33^, we do not use any measures related to pupil dilation, as the uncontrolled lighting conditions in real-world outdoor settings are a threat to the validity of the results achieved. Yet, the results we obtained by exploiting gaze features were still weak. One possible explanation for the particularly high confusion of classifying unfamiliar observations correctly may be a shift of attention: Empirical evidence by Young et al.^68^ suggests that increasing route familiarity results in an attention shift towards the surrounding environment. Similarly, by analyzing the visual attention of participants during wayfinding, Alinaghi and Giannopoulos^69^ found that familiar wayfinders are more aware of the local environment (as compared to unfamiliar participants). This awareness is not only reflected in their gaze behavior (fixations and saccades independent of the stimulus) but also in their visual attention to different types of areas of interest. The way we assessed familiarity by asking about familiar areas, POIs therein, and about a known route among two of these POIs maximizes familiarity of subjects. Based on the aforementioned evidence, it is, hence, likely that participants took the opportunity to look around while walking a familiar route. This behavior, however, can likely resemble the behavior of traveling through an unfamiliar environment, in which case “looking around” is needed to orient oneself (as opposed to participants walking in familiar environments who “look around” for hedonistic reasons). As a consequence, the model cannot appropriately distinguish the two different states of familiarity.

In contrast to our expectations, the combination of gaze and IMU features did not increase accuracy but resulted in a drop of accuracy of a few percent for both best-case and average-case. Comparing the confusion matrices of these IMU only and gaze+IMU best-case models, this drop can primarily be attributed to cases in which familiar observations were classified as unfamiliar ($0.0033\%$ in the IMU-only case as compared to $0.042\%$ in case of the combined feature set). Looking at the SHAP values, we see that only very few (two for the 80/20 split and three for the L5O4T splits) gaze-related features are among the 20 most important features, and all of them are dispersion related. The minimum fixation dispersion along the y axis is most important for the best-case model based on the L5O4T split. This feature is also most decisive when using the gaze data only. While the minimum fixation dispersion along the x-axis is ranked second in the case of using only gaze data, the same feature is ranked tenth, with yaw and acceleration features (in particular those along the IMU’s y-axis) being more important. The fact that dispersion-related features are important is in line with prior evidence indicating an impact of expertise-level on fixation dispersion (see, e.g., Duchowski^70^, p. 186, or Reingold et al.^71^).

These results also indicate that the importance of head and body movements is so strong that adding gaze-based features to IMU features does not usefully distinguish between a person walking through a familiar versus an unfamiliar environment. Obviously, the IMU features reflect a lot more than movements necessary for eye-head coordination, which are known to be key in visual cognitive processing in general^72^ and for the eye’s saccades in free head-movement scenarios like mobile eye-tracking in particular (see Freedman^73^ for a review of the impact of head movements on saccadic features, which we took into consideration when calculating the saccadic features).

We can identify at least three substantial limitations to our work. First, we conceptualized and measured familiarity as a binary variable, distinguishing only familiar and unfamiliar areas during the online data collection phase. While this binary distinction is widespread in the literature, a measurement comprising more than two levels would more precisely represent familiarity as a psychological variable and achieve better personalization for navigation systems. Second, we used auditory route instructions. While participants were given the opportunity to request these instructions as often as they wanted, we did not provide any further information or context to them, such as a map. We do, hence, not know whether our results hold across different route-instruction presentation modalities. Finally, using a single, head-mounted IMU makes it difficult to actually disentangle head rotations from rotations of other body parts, such as the upper body or even the full body. The rotation of different body parts may, in fact, be an indicator for different levels of familiarity: For example, it is plausible that in familiar environments, orientation can be maintained by moving only the head, whereas in unfamiliar environments, it might require a person to fully turn their body around.

## Conclusion and future work

We conducted a within-subject in-situ study in which we had pedestrian wayfinders find their way along two routes, one in a familiar environment and one in an unfamiliar environment. We applied machine-learning analyses of participants’ eye and head/body movements in order to classify them as familiar or unfamiliar with an environment. In doing so, we provided empirical evidence that IMU data is sufficient to distinguish these two levels of familiarity with an average accuracy of $89.9\%$. Yaw and the acceleration in left/right direction were very important features to achieve this accuracy; arguably both of these are primarily related to head movements. However, we also see evidence for large individual differences in body movements. In contrast to our expectations, gaze-based features alone did not yield good results, particularly not in classifying familiar datasets correctly. Moreover, mixing gaze-based features and IMU features actually negatively impacted the accuracy of classification (although very modestly at $4\%$ for the average case). Our main conclusion from these results is that gaze features (for example, obtained from smart glasses) will not help identify the wayfinder’s level of familiarity in order to personalize wayfinding instructions, but IMU features will quite effectively.

Finally, considering the limitations of our work discussed above, we intend to pursue three different avenues for future research. The first is to develop and investigate non-binary measures of spatial familiarity and measures more reflective of the variegated nature of familiarity. We will consider more than two levels of familiarity and use a richer and more complex approach to measuring familiarity, such as by asking multiple categories of self-report questions. Different questions will focus on different types of features, including landmarks, specific routes, and areas. Our second intention for future research is to administer route instructions of different styles and modes, instead of just auditory, landmark-based instructions. Finally, we will investigate classifying familiarity by assessing full-body movements. Full-body motion capture will not only help in understanding the impact of individual movement differences on classification accuracy; it may also provide further insights into people’s behavioral responses to different levels of familiarity, especially if familiarity is measured on an interval scale.

## Supplementary Information

Supplementary Information.

## Data Availability

The data as well as the analysis/machine learning scripts used for this work can be obtained through the research data repository of TU Wien (DOI: 10.48436/zjkky-pgs18) and from the website of the Geoinformation group (https://geoinfo.geo.tuwien.ac.at/resources/).
