# Supplementary material for: Predicting spatial familiarity by exploiting head and eye movements during pedestrian navigation in the real world
Source: Sci Rep. 2025 Mar 7;15:7970. doi: 10.1038/s41598-025-92274-4 (PMC11889111; doi:10.1038/s41598-025-92274-4)
Supplement: Supplementary file 1 — Supplementary Information. [file 41598_2025_92274_MOESM1_ESM.pdf]

Predicting spatial familiarity by exploiting head and eye movements during pedestrian navigation in the real world – Supplementary Information

Markus Kattenbeck[1,*], Ioannis Giannopoulos[1], Negar Alinaghi[1], Antonia Golab[2], and Daniel R. Montello[3]

[1]TU Wien, Research Unit Geoinformation, Vienna, 1040, Austria
[2]TU Wien, Energy Economics Group, Vienna, 1040, Austria
[3]UC Santa Barbara, Geography, Santa Barbara, CA 93117, USA
[*]markus.kattenbeck@tuwien.ac.at

February 22, 2025

Table 1: Overview of all 99 head-mounted IMU features.

Category	Features	Definition	Count
Statistical Measures (avg/ min/ max/ var)	Acc-X	Acceleration along the X axis	4
	Acc-Y	Acceleration along the Y axis	4
	Acc-Z	Acceleration along the Z axis	4
	Acc-all	Total acceleration, computed as the magnitude of the three acceleration signals	4
	Roll	The rotation around the longitudinal axis	4
	Pitch	The rotation around the lateral axis	4
	Yaw	The rotation around the vertical axis	4
Animals Movement Patterns	movement-intensity	The mean of the Euclidean norm (magnitude) of three-dimensional acceleration values	1
	signal-magnitude-area	The mean of the sum of the absolute values of three-dimensional acceleration components	1
	energy-acc	The mean of the squared power of the sum of the squares of three-dimensional acceleration components	1
	entropy-acc	The mean of the entropy-like measure based on the logarithm of the sum of the squares of the three-dimensional acceleration components	1
	movement-variation	The mean of the sum of the absolute differences between consecutive values of each of the three-dimensional acceleration components	1
Audio Signal Processing	acc-zero-crossing-count	The number of zero-crossings in the acceleration signal	1
	acc-zero-crossing-rate	The rate of zero-crossings in the acceleration signal	1
	acc-spectral-rolloff-mean	The rolloff point indicates the point in the frequency spectrum where most of the energy of the signal is concentrated. The mean and variance can be used to analyze the distribution of spectral energy in the signal.	1
	acc-spectral-rolloff-var		1
	acc-spectral-centroid	The spectral centroid is a measure that indicates where the center of mass of the spectrum is located.	1
	acc-chroma-frequencies-mean	The mean of the chroma features which represent the twelve different pitch classes	1
Human Movement Patterns	cepstral-coeff-Acc[0:50]	The average of scaled Mel-frequency cepstral coefficients (MFCCs). MFCC captures the timbral texture of the signal by representing its short-term power spectrum on a Mel frequency scale.	50
	p2p-Acc-X-amplitude	Peak-to-peak amplitude of the acceleration signals	1
	p2p-Acc-Y-amplitude		1
	p2p-Acc-Z-amplitude		1
	p2p-FreeAcc-E-amplitude		1
	p2p-FreeAcc-N-amplitude		1
	p2p-FreeAcc-U-amplitude		1
	acc-signal-frequency	The dominant frequency of a signal according to its autocorrelation function and identifying the delay corresponding to the first significant peak.	1
	avg-std-Acc	Average of the standard deviation of the three-dimensional acceleration components	1
	acc-fundamental-period	The mean period of zero-crossings in the acceleration signal, used to estimate the dominant frequency of the signal	1
Custom	freq-change-Yaw	The number of times the sequence of values in Yaw changes direction	1
